# Knowledge, attitude, and practice toward allergic rhinitis among parents in Ningbo, China

**DOI:** 10.1186/s12889-024-18581-z

**Published:** 2024-05-14

**Authors:** Lingya Lu, Jinyan Feng, Lihua Zhu, Aina Chen, Xuenai Chen, Yanming Lu

**Affiliations:** 1 Department of Pediatrics, Ningbo Hangzhou Bay Hospital, 315336 Ningbo, Zhejiang China; 2 Department of Pediatrics, Cixi Maternal and Child Health Hospital in Zhejiang Province, 315300 Cixi, China; 3 Ningbo Women and Children’s Hospital, 315012 Ningbo, Zhejiang China; 4 Department of Pediatrics, Renji Hospital, School of Medicine, Shanghai Jiaotong University, 201112 Shanghai, China

**Keywords:** Knowledge, attitude, practice, Children, Parents, Allergic rhinitis, Cross-sectional study

## Abstract

**Background:**

This study examined the knowledge, attitude, and practice (KAP) toward allergic rhinitis (AR) among parents.

**Methods:**

This cross-sectional study enrolled parents of children with AR at Ningbo Hangzhou Bay Hospital between December 2022 and March 2023. A self-administered questionnaire was developed to collect the demographic characteristics, knowledge, attitudes, and practices toward AR.

**Results:**

This study included 480 questionnaires, and 78.33% were mothers. The mean knowledge, attitude, and practice scores were 13.49 ± 6.62 (possible range: 0–24), 33.99 ± 3.40 (possible range: 8–40), and 21.52 ± 3.36 (possible range: 5–26), indicating poor knowledge, positive attitudes, and proactive practice. Multivariable logistic regression analysis showed living in urban areas in Ningbo outside Hangzhou Bay New Zone (OR = 4.33, 95%CI: 1.52–12.34, *P* = 0.006), living in rural areas in Ningbo (OR = 2.15, 95%CI: 1.00-4.59, *P* = 0.049), being self-employed (OR = 1.99, 95%CI: 1.00-3.95, *P* = 0.049), monthly income per capita ≥ 20,000 CNY (OR = 1.89, 95%CI: 1.02–3.47, *P* = 0.042), child with one biological sibling (OR = 0.48, 95%CI: 0.30–0.78, *P* = 0.003), and ≥ 6 times hospital visits for AR (OR = 2.32, 95%CI: 1.40–3.86, *P* = 0.001) were independently associated with adequate knowledge. The knowledge (OR = 1.09, 95%CI: 1.05–1.13, *P* < 0.001) and ≥ 6 times hospital visits for AR (OR = 1.84, 95%CI: 1.06–3.22, *P* = 0.032) were independently associated with a positive attitude. The knowledge (OR = 1.08, 95%CI: 1.04–1.13, *P* = 0.001), attitude (OR = 1.41, 95%CI: 1.28–1.55, *P* < 0.001), monthly income per capita ≥ 20,000 CNY (OR = 3.59, 95%CI: 1.49–8.65, *P* = 0.004), no previous hospital visit for AR (OR = 0.35, 95%CI: 0.16–0.78, *P* = 0.003), and ≥ 6 times hospital visits for AR (OR = 0.40, 95%CI: 0.20–0.81, *P* = 0.011) were independently associated with the practice scores.

**Conclusions:**

The parents of children with AR had poor knowledge but positive attitudes and proactive practice toward AR. This study has identified a need for specific and reliable information initiatives to be introduced as a means of reducing parental concern and ensuring evidence-based strategies for managing children with AR.

**Supplementary Information:**

The online version contains supplementary material available at 10.1186/s12889-024-18581-z.

## Background

Allergic rhinitis (AR) is a common type I hypersensitivity response of the upper respiratory tract to seasonal and perennial aeroallergens (e.g., grasses, outdoor mold spores, weeds, and trees), resulting in recurrent nasal congestion, rhinorrhea, sneezing, and mucosal itching of the nose, eyes, ears, and palate [1, 2]. AR is among the most common diagnoses in family medicine [3, 4]. The prevalence of AR varies widely (8.5-30%) among countries, regions, diagnostic criteria, and patient age groups [5, 6]. Seasonal AR is triggered by aeroallergens that vary based on location and climate [1, 2], while perennial AR is triggered by dust mites, indoor molds, animal dander, pollen in some climates, and occupational allergens [1]. The management of AR is based on allergen avoidance, intranasal corticosteroids, antihistamines, anticholinergics, nasal saline irrigation, and immunotherapy [2, 7–9]. Therefore, it is particularly important to enhance patients’ awareness and self-management of AR.

The knowledge, attitudes, and practice (KAP) methodology is a structured survey method that provides quantitative and qualitative data about the gaps, misunderstandings, and misconceptions that constitute barriers to adequately performing a specific subject in a specific population [10, 11]. KAP surveys are particularly useful to identify items that could be targeted in the future to improve the subject’s performance. Beyond the diagnosis of AR, the child and caregiver (e.g., the parents) are central in the self-management of AR since proper knowledge is essential to adopt adequate behaviors to decrease the attacks, take the proper medication on time, and improve prognosis [12]. The patients’ KAP on AR varies among countries from low to relatively good [13–17], but few studies focused on the parents of children with AR, and fewer studies were performed in China. Still, some studies revealed that the KAP of parents of children with asthma (a condition often associated with AR and more severe than simple AR [18]) was generally poor [19, 20].

Since children rely on their parents to help them manage diseases, evaluating the parents’ KAP is essential. Therefore, this study aimed to examine the KAP toward AR of parents of children with AR.

## Methods

### Study design and participants

This cross-sectional study enrolled parents of children (convenience sampling) with AR at Ningbo Hangzhou Bay Hospital between December 2022 and March 2023. The inclusion criteria were (1) parents of children aged 0–14, (2) settled down in Ningbo (China), and (3) their children were diagnosed with AR. In order to include as many people as possible and decrease the risk of selection bias, no exclusion criteria were set. This study was approved by the ethics committee of Ningbo Hangzhou Bay Hospital. All participants provided a signed informed consent form before completing the survey.

### Questionnaire

The questionnaire was designed based on the Chinese guidelines for the diagnosis and treatment of AR [21, 22], the guidelines for the diagnosis and treatment of pediatric AR, and the expert consensus on the diagnosis and treatment of children’s allergic diseases [23]. The first draft of the questionnaire was revised according to the comments of Prof. Lu Yanming, a member of the immunology Group of the Pediatric Branch of the Chinese Medical Association. A pre-test (53 samples) was performed and revealed that Cronbach’s α was 0.9266, indicating good internal consistency.

The final questionnaire included four dimensions with 46 items. Among them, the basic characteristics consisted of 17 items, the knowledge dimension consisted of 12 items, the attitude dimension consisted of 8 items, and the practice dimension consisted of 9 items. For the knowledge dimension, 2 points were scored for “well-known”, 1 point was scored for “partly known”, and 0 points were scored for “unknown”, with a score range was 0–24 points. The attitude dimension used a five-point Likert scale from very positive (5 points) to very negative (1 point) with a score range of 8–40 points. In the practice dimension, P1 investigated the treatment of AR in children, P2 investigated the measures taken by the parents when children suffered from AR, and P9 investigated ways for parents to learn about AR; these items only descriptive statistics. P3 investigated whether parents have taken their child for “allergen testing” with 1 point for “yes” and 0 points for “no” or “unclear”. P4-8 used a five-point Likert scale, rated from 5 points for very agree to 1 point for very disagree. The score range was 5–26 points. For all three dimensions, scores ≥ 70% of the total theoretical KAP scores were considered “adequate knowledge”, “positive attitude”, and “proactive practice” [24].

The participants were recruited during popularization activities of free diagnosis in and out of the hospital. The Wenjuanxing e-questionnaire platform (Wenjuanxing Tech Co., Ltd., Changsha, China) was used to create the electronic questionnaires and collect data. The QR code of the electronic questionnaire was provided to the participants in the consulting room, free diagnosis activities, and popular science activities. In order to avoid repetition, IP restriction was applied, which meant that the survey could only be completed once from a single IP address. All information was anonymously collected. The members of the research team were responsible for answering the participants’ questions in time. The research team conducted quality checks on the questionnaires. Obvious logical errors, such as the duration of the child’s first diagnosis of AR being greater than the child’s age or answering the same option for the whole questionnaire, were considered invalid questionnaires.

### Statistical analysis

Stata 17.0 (Stata Corporation, College Station, TX, USA) was used for statistical analysis. The continuous variables were expressed as mean ± standard deviation (SD) and analyzed using Student’s t-test (comparison between two groups) and ANOVA (comparison among multiple groups). The categorical variables were expressed as n (%) and analyzed using the chi-square test. Pearson’s correlation analysis was used to analyze the correlations between knowledge, attitude, and practice. Variables with *P* < 0.05 in the univariable logistic regression analysis were entered in the multivariable regression analysis. Multivariable logistic regression was performed to analyze the factors independently associated with the KAP. A confirmatory factor analysis was performed to evaluate the questionnaire’s validity and reliability. Two-tailed *P* < 0.05 was considered statistically significant.

## Results

### Characteristics of the participants

A total of 512 questionnaires were collected; 32 questionnaires with the same options or obvious logic errors were excluded. Therefore, 480 valid questionnaires (93.75%) were included for analysis. Most participants were mothers (78.33%), married (97.92%), with college or bachelor’s degree (64.38%), children in kindergarten (39.38%), employed (67.92%), with insurance for the children (85.20%), living in Hangzhou Bay New Zone (a district in Ningbo) (88.13%), and the primary caregiver of the child with AR (84.58%) (Table 1).

**Table 1:** Characteristics of the participants

Variables	n (%)	Knowledge scores		Attitude scores		Practice scores	
		Mean ± SD	P	Mean ± SD	P	Mean ± SD	P
**Total scores**	480	13.49 ± 6.62		33.99 ± 3.40		21.52 ± 3.36	
**Age**			0.911		0.512		0.735
24–35	191 (39.79)	13.46 ± 6.52		34.20 ± 3.23		21.49 ± 3.29	
36–40	175 (36.46)	13.38 ± 6.74		33.90 ± 3.42		21.42 ± 3.46	
> 40	114 (23.75)	13.72 ± 6.65		33.77 ± 3.65		21.73 ± 3.34	
**Age of child**			0.010		0.031		0.338
≤ 6 (preschool)	190 (40.25)	12.82 ± 6.48		33.94 ± 3.28		21.23 ± 3.36	
7–10	93 (19.70)	15.27 ± 6.27		34.75 ± 2.74		21.81 ± 3.33	
11–17	189 (40.04)	13.17 ± 6.73		33.62 ± 3.73		21.59 ± 3.37	
**Gender of child**			0.264		0.776		0.367
Male	288 (60.00)	13.77 ± 6.50		34.03 ± 3.22		21.63 ± 3.26	
Female	192 (40.00)	13.08 ± 6.79		33.94 ± 3.66		21.35 ± 3.52	
**Relationship with child**			0.800		0.266		0.148
Father	104 (21.67)	13.35 ± 7.24		33.66 ± 3.91		21.10 ± 3.33	
Mother	376 (78.33)	13.53 ± 6.45		34.08 ± 3.24		21.64 ± 3.37	
**Marital status**			0.560		0.067		0.214
Married	470 (97.92)	13.46 ± 6.58		33.94 ± 3.40		21.49 ± 3.37	
Divorced	9 (1.88)	14.44 ± 8.99		36.00 ± 2.55		22.78 ± 2.99	
Widowed	1 (0.21)	20.00		39.00		26.00	
**Residence**			< 0.001		0.529		0.170
Hangzhou Bay New Zone	423 (88.13)	13.00 ± 6.56		33.94 ± 3.41		21.42 ± 3.36	
Urban areas in Ningbo City outside Hangzhou Bay New Zone	21 (4.38)	18.24 ± 5.45		34.76 ± 3.05		22.62 ± 3.61	
Rural areas in Ningbo City	36 (7.50)	16.56 ± 6.15		34.17 ± 3.55		22.06 ± 3.23	
**Education**			0.070		0.098		0.125
Junior high school	59 (12.29)	11.54 ± 7.8		33.81 ± 4.02		21.17 ± 3.61	
High school/technical secondary school	89 (18.54)	13.08 ± 6.64		34.76 ± 3.38		22.26 ± 3.14	
College/bachelor’s	309 (64.38)	13.95 ± 6.37		33.86 ± 3.28		21.41 ± 3.40	
Master’s or above	23 (4.79)	13.87 ± 7.50		33.26 ± 3.00		21.04 ± 2.84	
**Educational of the child**			0.002		0.026		0.206
Not yet enrolled	11 (2.29)	14.27 ± 6.94		34.18 ± 4.00		20.55 ± 2.91	
Kindergarten	189 (39.38)	12.73 ± 6.36		33.92 ± 3.21		21.23 ± 3.36	
Elementary school	108 (22.50)	15.58 ± 6.49		34.81 ± 2.91		21.99 ± 3.35	
Junior high school	172 (35.83)	12.97 ± 6.74		33.55 ± 3.77		21.60 ± 3.38	
**Working status**			0.008		0.858		0.258
Employed	326 (67.92)	13.54 ± 6.67		33.89 ± 3.47		21.34 ± 3.42	
Unemployed	9 (1.88)	12.33 ± 7.71		33.78 ± 2.95		21.89 ± 3.06	
Individual business	56 (11.67)	15.84 ± 6.41		34.34 ± 3.23		22.38 ± 3.18	
Full-time wife/husband	88 (18.33)	12.05 ± 6.05		34.19 ± 3.35		21.63 ± 3.26	
Others	1 (0.21)	4.00		33.00		19.00	
**Monthly income per capita, CNY**			0.006		0.651		0.082
< 2000	16 (3.33)	10.94 ± 6.50		33.63 ± 4.35		21.75 ± 2.89	
2000–4999	79 (16.46)	12.29 ± 7.09		33.75 ± 3.61		21.37 ± 3.71	
5000–9999	179 (37.29)	12.81 ± 6.27		33.82 ± 3.50		21.02 ± 3.52	
10,000–19,999	116 (24.17)	14.78 ± 6.37		34.34 ± 2.78		21.94 ± 3.01	
≥ 20,000	90 (18.75)	14.69 ± 6.83		34.17 ± 3.56		22.06 ± 3.13	
**Health insurance of child**			–		–		–
Children’s medical insurance	393 (81.88)	13.64 ± 6.52		34.12 ± 3.33		21.59 ± 3.36	
Commercial insurance	102 (21.25)	14.18 ± 6.42		34.24 ± 3.33		22.03 ± 3.16	
No insurance	33 (6.88)	12.79 ± 8.00		32.91 ± 4.40		21.21 ± 3.68	
Others	38 (7.92)	12.03 ± 6.77		32.95 ± 3.69		20.47 ± 3.32	
**Number of child’s biological siblings**			0.024		0.953		0.787
0	174 (36.25)	14.54 ± 6.24		33.99 ± 3.20		21.38 ± 3.20	
1	224 (46.67)	12.71 ± 6.77		34.03 ± 3.57		21.58 ± 3.41	
≥ 2	82 (17.08)	13.39 ± 6.76		33.89 ± 3.37		21.63 ± 3.61	
**Duration of disease in child**			0.013		0.095		0.002
< 1 year	99 (20.63)	11.75 ± 6.63		33.60 ± 3.47		20.85 ± 3.32	
1-2.9 years	163 (33.96)	13.42 ± 6.30		34.07 ± 3.23		21.43 ± 3.27	
3-4.9 years	118 (24.58)	14.53 ± 6.99		34.57 ± 3.30		22.47 ± 3.17	
≥ 5 years	100 (20.83)	14.11 ± 6.42		33.57 ± 3.66		21.21 ± 3.59	
**Number of hospital visits for AR**			< 0.001		< 0.001		< 0.001
0 times	53 (11.04)	11.42 ± 6.43		33.74 ± 3.56		20.04 ± 3.33	
1–5 times	285 (59.38)	12.80 ± 6.46		33.69 ± 3.39		21.47 ± 3.24	
≥ 6 times	142 (29.58)	15.66 ± 6.50		35.06 ± 3.10		22.18 ± 3.46	
**Primary caregiver for the child**			0.751		0.215		0.031
Yes	406 (84.58)	13.45 ± 6.64		34.07 ± 3.38		21.66 ± 3.35	
No	74 (15.42)	13.72 ± 6.52		33.54 ± 3.48		20.74 ± 3.33	
**Other diseases (child)**			< 0.001		0.068		0.600
Asthma	59 (12.29)	16.81 ± 5.86		34.81 ± 2.76		22.00 ± 3.37	
Atopic dermatitis	27 (5.63)	15.41 ± 6.89		34.70 ± 3.10		21.89 ± 3.45	
Eczema	123 (25.63)	12.84 ± 6.51		33.51 ± 3.39		21.40 ± 3.18	
Other	271 (56.46)	12.87 ± 6.58		33.96 ± 3.53		21.43 ± 3.44	

### Knowledge, attitude, and practice

The mean knowledge score was 13.49 ± 6.62 (possible range: 0–24), indicating poor knowledge (162 of 480 participants had good knowledge). Higher knowledge scores were observed in parents of children of 7–10 years (*P* = 0.010), living in Urban areas in Ningbo outside Hangzhou Bay New Zone (*P* < 0.001), children in elementary school (*P* = 0.002), self-employed (*P* = 0.008), only child (*P* = 0.024), with a longer history of AR (*P* = 0.013), with ≥ 6 hospital visits for AR (*P* < 0.001), and with a co-diagnosis of asthma (*P* < 0.001) (Table 1). The item with the highest rate of well-known/little understanding was K2 (89.58%; “The typical symptoms of allergic rhinitis in children are watery rhinorrhea, nasal itching, nasal congestion, and sneezing”), and the item with the lowest understanding was K8 (39.96%; “Children with allergic rhinitis and concurrent attacks of persistent bronchial asthma cannot receive desensitization therapy”) (Table 2).

**Table 2:** Knowledge

Knowledge	Well-known, n (%)	Partly known, n (%)	Unknown, n (%)
K1. Children’s allergic rhinitis is a non-infectious chronic inflammatory disease of the nasal mucosa after exposure to allergens such as dust mites and pollen; do you know?	187 (38.96)	240 (50)	53 (11.04)
K2. The typical symptoms of allergic rhinitis in children are watery rhinorrhea, nasal itching, nasal congestion, and sneezing; do you know?	223 (46.46)	207 (43.13)	50 (10.42)
K3. When a primary family member has an allergic disease, the child should be managed as a child at high risk of allergic disease; do you know?	159 (33.13)	191 (39.79)	130 (27.08)
K4. Allergens refer to antigen substances that induce the body to produce allergies. Most antigens are inhaled antigens; dust mites and pollen are the most common; do you know?	216 (45)	199 (41.46)	65 (13.54)
K5. Inhalation of antigens such as mold, animal dander, and cockroaches may also cause allergic rhinitis attacks in children; do you know?	198 (41.25)	192 (40)	90 (18.75)
K6. Do you know about allergen skin testing?	156 (32.5)	152 (31.67)	172 (35.83)
K7. Do you know about desensitization therapies (such as dust mite drops) for allergic rhinitis in children?	116 (24.17)	146 (30.42)	218 (45.42)
K8. Children with allergic rhinitis and concurrent attacks of persistent bronchial asthma cannot receive desensitization therapy; do you know?	92 (19.17)	95 (19.79)	293 (61.04)
K9. Children with allergic rhinitis should avoid or minimize exposure to allergens; do you know?	264 (55)	157 (32.71)	59 (12.29)
K10. Do you know effective measures to control indoor dust mites?	207 (43.13)	207 (43.13)	66 (13.75)
K11. Allergic diseases include food allergies, atopic dermatitis, allergic rhinitis, and allergic asthma; do you know?	173 (36.04)	219 (45.63)	88 (18.33)
K12. One child may have multiple allergic diseases simultaneously; do you know?	158 (32.92)	173 (36.04)	149 (31.04)

The mean attitude score was 33.99 ± 3.40 (possible range: 8–40), indicating positive attitudes (364 of 480 participants had positive attitudes). Higher attitude scores were observed in parents of children of 7–10 years (P = 0.031), children in elementary school (P = 0.026), and ≥ 6 hospital visits for AR (P < 0.001) (Table 1). The item with the highest positive responses was A2 (93.90%; You are concerned that allergic rhinitis may endanger your child’s health.”). The item with the lowest positive responses was A5 (45.88%; “You think your child’s allergic rhinitis is currently well controlled and does not adversely affect his/her daily life.”) (Table 3).

**Table 3:** Attitude, n (%)

	Strongly agree	Agree	Neutral	Disagree	Strongly disagree
A1. How much do you care about allergic rhinitis in your child:	162 (58.06)	95 (34.05)	19 (6.81)	3 (1.08)	0
A2. You are concerned that allergic rhinitis may endanger your child’s health.	170 (60.93)	92 (32.97)	16 (5.73)	1 (0.36)	0
A3. You think allergic rhinitis will be relieved on its own, so there is no need to pay attention to it.	12 (4.3)	12 (4.3)	55 (19.71)	83 (29.75)	117 (41.94)
A4. You think that allergic rhinitis in children needs to be treated with standardized medical protocol	178 (63.8)	74 (26.52)	26 (9.32)	0	1 (0.36)
A5. You think your child’s allergic rhinitis is currently well controlled and does not adversely affect his/her daily life.	43 (15.41)	85 (30.47)	67 (24.01)	59 (21.15)	25 (8.96)
A6. How important do you think it is to change clothes and wash towels and bedding frequently:	195 (69.89)	78 (27.96)	5 (1.79)	1 (0.36)	0
A7. Exercise and strengthening physical fitness are beneficial to prevent the onset of allergic rhinitis in children	215 (77.06)	57 (20.43)	7 (2.51)	0	0
A8. For a child with allergic rhinitis, you will worry that he is more likely to develop asthma than the average child.	123 (44.09)	112 (40.14)	29 (10.39)	11 (3.94)	4 (1.43)

The mean practice score was 21.52 ± 3.36 (possible range: 6–25), indicating sufficient practice (385 of 480 participants had proactive practice). Higher practice scores were observed in parents of children with a history of AR of 3-4.9 years (*P* = 0.002), ≥ 6 hospital visits for AR (*P* < 0.001), and the primary caregiver of the child (*P* = 0.031) (Table 1). The item with the highest positive responses was P5 (91.88%; “You will regularly wash your child’s pillow towels, sheets, toys, and other items.”), The item with the lowest positive responses was P8 (62.91%; “You will teach your child about allergic rhinitis.”) (Table 4).

**Table 4:** Practice, n (%)

	Strongly agree	Agree	Neutral	Disagree	Strongly disagree
P4. You will supervise and teach your child to avoid exposure to allergens.	205 (42.71)	177 (36.88)	87 (18.13)	9 (1.88)	2 (0.42)
P5. You will regularly wash your child’s pillow towels, sheets, toys, and other items.	294 (61.25)	147 (30.63)	37 (7.71)	1 (0.21)	1 (0.21)
P6. You will guide your child to take active physical exercise.	260 (54.17)	169 (35.21)	50 (10.42)	1 (0.21)	0
P7. You will actively learn about allergic rhinitis.	167 (34.79)	154 (32.08)	140 (29.17)	16 (3.33)	3 (0.63)
P8. You will teach your child about allergic rhinitis.	154 (32.08)	148 (30.83)	148 (30.83)	24 (5)	6 (1.25)
	Yes	No	Unclear		
P3. Whether the child has been tested for allergens?	249 (51.88)	200 (41.67)	31 (6.46)		

**Fig. 1:**
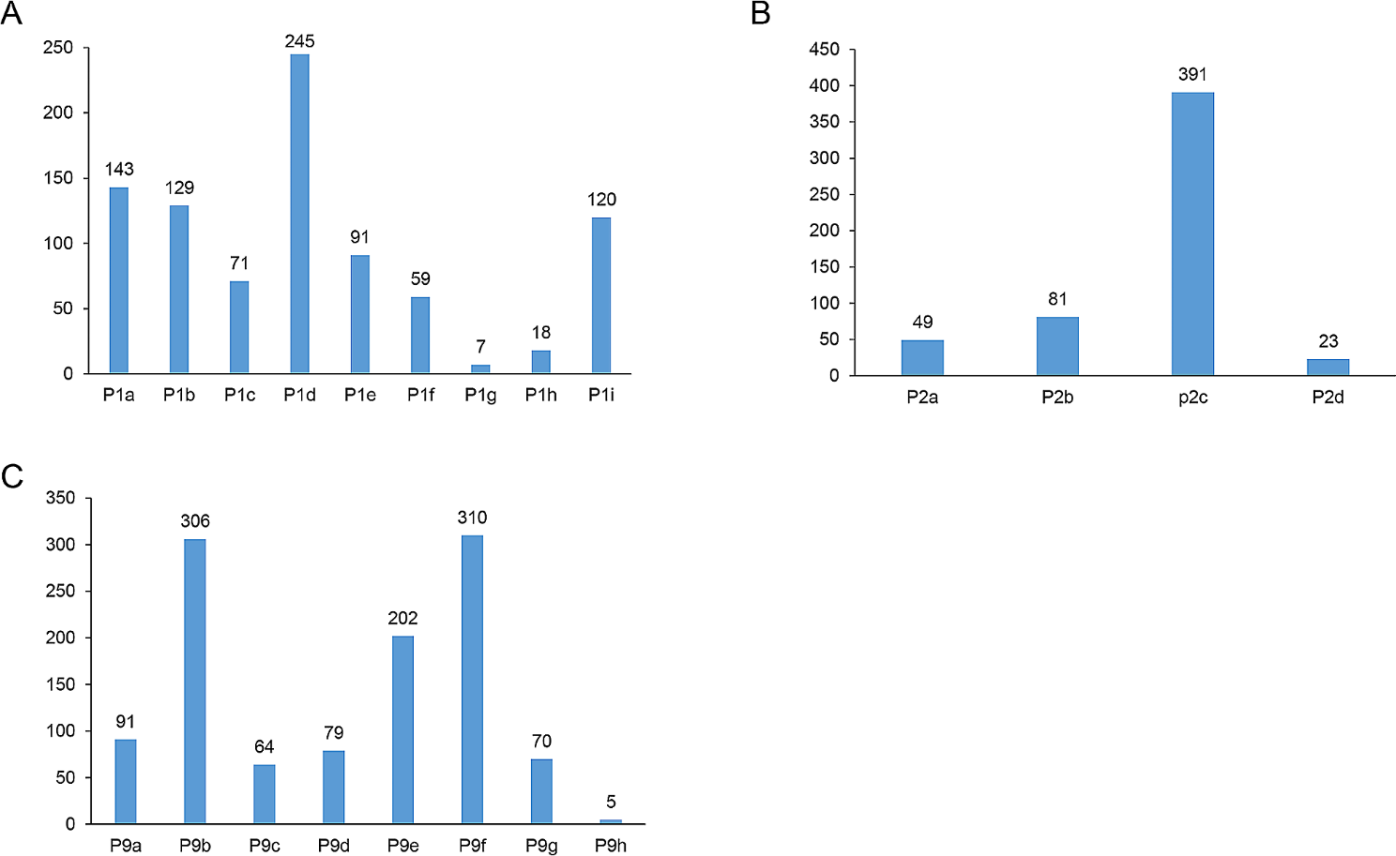
(**A**) How is your child treated for allergic rhinitis (multiple choice)? (a) Oral antihistamines; (b) Nasal antihistamines; (c) Nasal glucocorticoids; (d) Nasal irrigation with saline or seawater; (e) Chinese medicine; (f) Immunotherapy; (g) Surgery; (h) Other treatments; (i) I don’t know. (**B**) What parents do when their child suffers from allergic rhinitis (multiple choice) (a) No treatment, wait for its own relief; (b) Self-dispensing; (c) Go to the hospital and receive proper treatment; (d) Others. (**C**) How do you learn about allergic rhinitis? (a) Community advocacy; (b) Internet; (c) Magazines; (d) Television broadcasting; (e) Communication among friends; (f) Introduction of medical staff; (g) Others; (h) No care about such information

Most children were tested for allergens (51.88%). The most common treatments include nasal irrigation, oral antihistamines, and nasal antihistamines (Fig. 1A). Most parents go to the hospital for treatment when their child suffers from AR (Fig. 1B). Most parents learned about AR from medical staff, the Internet, and communication with friends (Fig. 1C**)**. Supplementary Table S1 showed the KAP item comparisons between the mothers and fathers.

### Correlations

As shown in Supplementary Table S2, the knowledge scores were correlated to the attitude (*r* = 0.310, *P* < 0.001) and practice (*r* = 0.477, *P* < 0.001) scores. The attitude scores were correlated to the practice scores (*r* = 0.551, *P* < 0.001).

### Multivariable analysis

Multivariable logistic regression analysis showed living in urban areas in Ningbo outside Hangzhou Bay New Zone (OR = 4.33, 95%CI: 1.52–12.34, *P* = 0.006), living in rural areas in Ningbo (OR = 2.15, 95%CI: 1.00-4.59, *P* = 0.049), being self-employed (OR = 1.99, 95%CI: 1.00-3.95, *P* = 0.049), monthly income per capita ≥ 20,000 CNY (OR = 1.89, 95%CI: 1.02–3.47, *P* = 0.042), child with one biological sibling (OR = 0.48, 95%CI: 0.30–0.78, *P* = 0.003), and ≥ 6 times hospital visits for AR (OR = 2.32, 95%CI: 1.40–3.86, *P* = 0.001) were independently associated with adequate knowledge. The knowledge (OR = 1.09, 95%CI: 1.05–1.13, *P* < 0.001) and ≥ 6 times hospital visits for AR (OR = 1.84, 95%CI: 1.06–3.22, *P* = 0.032) were independently associated with a positive attitude. The knowledge (OR = 1.08, 95%CI: 1.04–1.13, *P* = 0.001), attitude (OR = 1.41, 95%CI: 1.28–1.55, *P* < 0.001), monthly income per capita ≥ 20,000 CNY (OR = 3.59, 95%CI: 1.49–8.65, *P* = 0.004), no previous hospital visit for AR (OR = 0.35, 95%CI: 0.16–0.78, *P* = 0.003), and ≥ 6 times hospital visits for AR (OR = 0.40, 95%CI: 0.20–0.81, *P* = 0.011) were independently associated with the practice scores (Table 5).

**Table 5:** Univariable and multivariable logistic regression analysis

Dependent variables	Independent variables	Univariable analysis		Multivariable analysis	
		OR (95%CI)	P	OR (95%CI)	P
**Knowledge**	**Age of the child**				
	≤ 6 (preschool)	Ref.		Ref.	
	7–10	2.02 (1.21, 3.38)	0.007	1.12 (0.61, 2.09)	0.710
	11–17	1.21 (0.78, 1.86)	0.390	0.84 (0.46, 1.52)	0.562
	**Residence**				
	Hangzhou Bay New Zone	Ref.		Ref.	
	Urban areas in Ningbo City outside Hangzhou Bay New Zone	5.76 (2.19, 15.19)	< 0.001	4.33 (1.52, 12.34)	0.006
	Rural areas in Ningbo City	2.58 (1.30, 5.12)	0.007	2.15 (1.00, 4.59)	0.049
	**Educational**				
	Junior high school	0.49 (0.25, 0.95)	0.034	0.70 (0.33, 1.50)	0.361
	High school/technical secondary school	0.72 (0.43, 1.19)	0.201	0.80 (0.44, 1.44)	0.447
	College/bachelor’s	Ref.		Ref.	
	Master’s or above	1.33 (0.57, 3.14)	0.509	1.01 (0.39, 2.62)	0.985
	**Working status**				
	Employed	Ref.		Ref.	
	Unemployed	0.92 (0.23, 3.74)	0.904	1.91 (0.41, 8.85)	0.407
	Individual business	1.48 (0.83, 2.63)	0.181	1.99 (1.00, 3.95)	0.049
	Full-time wife/husband	0.51 (0.29, 0.88)	0.016	0.61 (0.33, 1.13)	0.117
	Others	Omitted		Omitted	
	**Monthly income per capita, CNY**				
	< 2000	0.38 (0.08, 1.73)	0.210	0.50 (0.10, 2.50)	0.403
	2000–4999	1.09 (0.61, 1.96)	0.774	1.28 (0.68, 2.43)	0.441
	5000–9999	Ref.	0.006	Ref.	
	10,000–19,999	2.01 (1.23, 3.29)	0.015	1.71 (0.99, 2.94)	0.053
	≥ 20,000	1.94 (1.14, 3.30)		1.89 (1.02, 3.47)	0.042
	**Number of child’s biological siblings**				
	0	Ref.		Ref.	
	1	0.57 (0.37, 0.86)	0.008	0.48 (0.30, 0.78)	0.003
	≥ 2	0.66 (0.38, 1.15)	0.139	0.67 (0.36, 1.27)	0.222
	**Duration of AR in child**				
	< 1 year	Ref.		Ref.	
	1-2.9 years	1.50 (0.85, 2.67)	0.162	1.35 (0.72, 2.56)	0.351
	3-4.9 years	2.52 (1.39, 4.55)	0.002	2.00 (0.95, 4.21)	0.069
	≥ 5 years	1.94 (1.05, 3.60)	0.036	1.53 (0.65, 3.60)	0.332
	**Number of hospital visits for AR**				
	0 times	0.75 (0.37, 1.50)	0.416	0.79 (0.36, 1.70)	0.541
	1–5 times	Ref.		Ref.	
	≥ 6 times	2.49 (1.64, 3.79)	< 0.001	2.32 (1.40, 3.86)	0.001
**Attitude**	**Knowledge**	1.10 (1.06, 1.14)	< 0.001	1.09 (1.05, 1.13)	< 0.001
	**Relationships with child**				
	Father	0.61 (0.38, 0.98)	0.043	0.62 (0.37, 1.02)	0.061
	Mother	Ref.		Ref.	
	**Number of hospital visits for AR**				
	0 times	0.73 (0.39, 1.37)	0.330	0.79 (0.41, 1.51)	0.472
	1–5 times	Ref.		Ref.	
	≥ 6 times	2.30 (1.34, 3.94)	0.003	1.84 (1.06, 3.22)	0.032
**Practice**	**Knowledge**	1.11 (1.07, 1.16)	< 0.001	1.08 (1.04, 1.13)	0.001
	**Attitude**	1.40 (1.30, 1.52)	< 0.001	1.41 (1.28, 1.55)	< 0.001
	**Monthly income per capita, CNY**				
	< 2000	2.35 (0.51, 10.74)	0.270	5.12 (0.86, 30.36)	0.072
	2000–4999	1.06 (0.57, 1.96)	0.852	1.21 (0.58, 2.54)	0.604
	5000–9999	Ref.		Ref.	
	10,000–19,999	1.83 (1.00, 3.35)	0.051	1.35 (0.69, 2.66)	0.381
	≥ 20,000	2.41 (1.18, 4.93)	0.016	3.59 (1.49, 8.65)	0.004
	**Duration of AR in child**				
	< 1 year	Ref.		Ref.	
	1-2.9 years	1.36 (0.75, 2.49)	0.315	0.88 (0.42, 1.84)	0.730
	3-4.9 years	2.04 (1.01, 4.11)	0.046	1.47 (0.62, 3.49)	0.383
	≥ 5 years	1.01 (0.53, 1.94)	0.968	1.03 (0.42, 2.51)	0.949
	**Number of hospital visits for AR**				
	0 times	0.38 (0.20, 0.72)	0.003	0.35 (0.16, 0.78)	0.01
	1–5 times	Ref.		Ref.	
	≥ 6 times	0.95 (0.56, 1.60)	0.845	0.40 (0.20, 0.81)	0.011

### Subgroup analysis according to the children’s age

As shown in Supplementary Table S3, the parental KAP was significantly different among children’s age groups for K3 (*P* = 0.049), K4 (*P* = 0.050), K6 (*P* = 0.001), K7 (*P* = 0.001), K8 (*P* = 0.015), K9 (*P* = 0.010), A1 (*P* = 0.002), A2 (*P* < 0.001), A4 (*P* = 0.030), A8 (*P* = 0.012), and P8 (*P* = 0.018), with the highest scores being observed for the 7–10 age group, except for P8, for which the scores were the highest for the 7–10 and 11–17 age groups.

### Confirmatory factor analysis

The results of the confirmatory factor analysis (Supplementary Figure S1) showed that the CFI was 0.833 (> 0.800 is good), the IFI was 0.834 (> 0.800 is good), the TLI was 0.817 (> 0.800 is good), and the CMIN/DF was 4.367 (> 1; 1–3 is excellent, 3–5 is good)), indicating that the questionnaire has good reliability.

## Discussion

The results suggest that the parents of children with AR had poor knowledge but positive attitudes and proactive practice toward AR. Residence, biological sibling, and hospital visits for AR were independently associated with adequate knowledge. Knowledge and hospital visits for AR were independently associated with a positive attitude. Knowledge, attitude, monthly income per capita, and hospital visits for AR were independently associated with proactive practice. It is necessary to enhance education for parents in specific conditions.

Besides medication (which constitutes the second treatment used by the children after nasal irrigation in the present study), managing AR involves avoiding the identified allergens (when identified, since only about half of the children had been tested in the present study) and having good life habits [2, 7–9]. Therefore, a high KAP level plays a major role in the management of AR, but children, especially young children, can have difficulties in self-management because of immature knowledge and attitudes, especially in the face of a non-lethal allergy causing only respiratory symptoms. The present study revealed poor knowledge but positive attitudes and sufficient practice toward AR of parents of children with AR. It suggests that the parents are willing to take care of their children, improve their condition, and perform adequate actions to achieve that goal but are missing knowledge about why they pose specific actions. It could be because they are performing some actions out of habit or applying instructions they heard from the healthcare providers or read somewhere but without understanding why they do it. The knowledge and attitude scores were independently associated with the practice scores. Therefore, the results suggest that even though the knowledge levels were low, the participants were active toward AR out of habit or following medical advice but without understanding it. Nevertheless, due to the direct correlations and independent associations, improving knowledge should also improve attitudes and practice. Therefore, educational interventions (e.g., posters, pamphlets, video capsules, podcasts, etc.) should be created to improve the KAP of patients with AR. Of note, poor scores were observed for knowledge items related to the indications/contraindications of desensitization therapy, allergy skin testing, and the possibility of multiple allergic diseases simultaneously. Knowledge about those items should be enforced, but knowledge pertaining to the other items was not perfect either and should be improved.

Previous studies generally support the present one and show variable KAP in patients with AR [13–17, 25, 26]. Bhargave et al. [13] revealed large discrepancies among countries regarding the KAP of patients and physicians toward AR. In Saudi Arabia, the KAP of patients with AR was low [14, 16]. Similar results were reported in India [15] and four Southeast Asian nations [17]. Thai patients have poor knowledge of the risks of immunotherapy for AR [25], and similar results were observed in German athletes with AR [26]. Some of these studies also included healthcare providers, who also showed relatively poor or moderate KAP. Hence, there is a need to improve the KAP toward AR, especially in healthcare providers, since they are an important source of information for patients, as shown in the present study. Still, these previous studies were not performed specifically on parents of children with AR (although it can be considered that many of the participants in those previous studies had children), and no data are available in the literature regarding the specific population of parents of children with AR. In the present study, 12.29% of the patients had comorbid asthma, and AR is a risk factor for asthma exacerbations [27]. The knowledge scores of parents of children with comorbid AR and asthma were the highest, probably due to the diagnosis of asthma, which is more severe than a diagnosis of AR. Still, the KAP of parents of children with asthma was generally poor in China, including the items on AR [19, 20]. The present study showed that parents with another child besides the one with AR had a lower level of knowledge, perhaps because they had less time to gain knowledge. A history of more hospital visits for AR was associated with higher scores, probably because of more opportunities to gain knowledge from the medical staff, which was also reported by the participants as their main source of AR information. Living in rural areas was associated with lower knowledge scores. Disparities in healthcare services and health literacy between urban and rural residents in China are well-known [28–30]. Rural areas are vast and less densely populated than urban areas, and most of the healthcare services in rural areas are clinics and primary hospitals. The socioeconomic level is also usually lower in rural areas, and the socioeconomic level is a determinant of health literacy [31]. Education was not independently associated with knowledge in the present study, possibly because of smaller numbers of participants with lower education or lower income or possibly because urban/rural residence was covariant with education and income. A higher income was associated with better knowledge, attitudes, and practice, probably because of a higher capacity to pay for medical visits and treatments. No previous hospital visits for AR were associated with a low practice score, which supports the idea that the parents follow medical advice to help manage AR in their children; indeed, participants who did not have the opportunity to receive advice cannot apply it.

In the present study, the participants were most likely to be the children’s mothers. The subgroup analysis showed that mothers had higher scores than fathers regarding the attention that should be paid to AR, the importance of washing clothes, towels, and bedding, and the importance of physical activity, while the fathers had higher scores regarding the knowledge of desensitization therapy. It is well known that the mother’s and father’s attitudes toward a child’s care are different [32–34]. Still, the mother/father relationship with the children was not independently associated with the KAP dimensions. It could be related to the fact that not all mothers and fathers completed the questionnaire, but only those who attended the clinic and fathers going to the clinic for their children might have a more positive attitude toward the health of their children than others. It will have to be examined in future studies.

The results of the subgroup analysis based on children’s age suggest that knowledge was higher and attitudes were more positive when the children entered school and that the practice of teaching the child about AR is higher in school-age children and teenagers. It could be related to the children being more able to understand the disease and instructions about it.

This study has limitations. It was performed at a single center, and the resulting sample size was relatively small, considering the high prevalence of AR. The participants were primarily from Hangzhou Bay New Zone, a district in Ningbo. As a result, the findings may not fully represent Ningbo City. Additionally, the participants’ socioeconomic status was relatively elevated, which does not represent the general Chinese population. It was a cross-sectional study, and causality could not be investigated. Still, the results could serve as a baseline to examine the impact of future educational interventions. There were some differences between mothers and fathers regarding some KAP items, but the over-representation of mothers can bias the results. Finally, all KAP studies are at risk of social desirability bias [35, 36], which can overestimate the scores because some participants might be tempted to answer what they know they should do instead of what they are doing. Since the attitude and practice scores were high, that bias is possible.

## Conclusions

In conclusion, the parents of children with AR had poor knowledge but positive attitudes and sufficient practice toward AR. This study identified gaps in knowledge that would warrant future educational interventions. Improving knowledge should translate into more positive attitudes and more active practice.

### Electronic supplementary material

Below is the link to the electronic supplementary material.

Supplementary Material 1

## Data Availability

All data generated or analyzed during this study are included in this article and its supplementary information file.

## Abbreviations

KAP: knowledge Attitude and Practices
AR: Allergic Rhinitisx
SD: Standard Deviation
