# Supplementary material for: Knowledge, attitude, and practice toward allergic rhinitis among parents in Ningbo, China
Source: BMC Public Health. 2024 May 14;24:1310. doi: 10.1186/s12889-024-18581-z (PMC11092245; doi:10.1186/s12889-024-18581-z)

Supplementary Table S1. Parents’ KAP scores

| KAP items | Score, mean ± SD | | P |
| --- | --- | --- | --- |
|  | Father | Mother |  |
| K1. Children’s allergic rhinitis, also known as children’s allergic rhinitis, is a non-infectious chronic inflammatory disease of the nasal mucosa after exposure to allergens such as dust mites and pollen. | 1.22±0.71 | 1.30±0.63 | 0.305 |
| K2. The typical symptoms of allergic rhinitis in children are watery discharge, nasal itching, nasal congestion, and sneezing. | 1.26±0.70 | 1.39±0.65 | 0.080 |
| K3. When a primary family member has an allergic disease, the child should be managed as a child at high risk of allergic disease. | 1.03±0.78 | 1.07±0.77 | 0.639 |
| K4. Allergens refer to antigen substances that induce the body to produce allergies, mostly inhaled antigens, with dust mites and pollen being the most common. | 1.28±0.74 | 1.32±0.69 | 0.556 |
| K5. Inhaled antigens such as mold, animal dander, and cockroaches may also cause allergic rhinitis attacks in children. | 1.19±0.75 | 1.23±0.74 | 0.612 |
| K6. Do you know about allergen skin testing? | 1.04±0.80 | 0.95±0.83 | 0.318 |
| K7. Do you know about desensitization therapies (such as dust mite drops) for allergic rhinitis in children? | 0.87±0.83 | 0.77±0.80 | 0.267 |
| K8. Children with allergic rhinitis and persistent bronchial asthma cannot receive desensitization therapy. | 0.76±0.84 | 0.53±0.77 | 0.009 |
| K9. Children with allergic rhinitis should avoid contact or minimize contact with allergens. | 1.34±0.75 | 1.45±0.69 | 0.137 |
| K10. Effective measures to control indoor dust mites: keep the room clean, air circulation, reduce humidity, regularly clean the air conditioning filter; use well-sealed bed boards and pillows; wash bed sheets and pillows with hot water > 54.4 °C; Stay away from stuffed animals, carpets, tapestries, etc. | 1.19±0.74 | 1.32±0.68 | 0.093 |
| K11. Allergic diseases include food allergies, atopic dermatitis, allergic rhinitis, and allergic asthma. | 1.13±0.74 | 1.19±0.71 | 0.495 |
| K12. One child may suffer from multiple allergic diseases at the same time. | 1.04±0.80 | 1.01±0.80 | 0.777 |
| A1. How much do you care about allergic rhinitis in your child: | 4.38±0.69 | 4.38±0.72 | 0.946 |
| A2. You are concerned that allergic rhinitis may endanger your child’s health. | 4.38±0.73 | 4.46±0.69 | 0.256 |
| A3. Allergic rhinitis in children will relieve itself, there is no need to pay much attention. | 3.66±1.28 | 3.94±1.08 | 0.028 |
| A4. Allergic rhinitis in children requires standardized medical visits and treatment. | 4.51±0.70 | 4.45±0.72 | 0.490 |
| A5. The child’s allergic rhinitis is currently well-controlled and has not adversely affected his daily life. | 3.49±1.16 | 3.21±1.19 | 0.032 |
| A6. The importance of changing clothes frequently, washing towels and bedding frequently: | 4.55±0.62 | 4.70±0.50 | 0.010 |
| A7. For exercise, strengthening physical fitness is beneficial to prevent the onset of allergic rhinitis in children | 4.56±0.65 | 4.73±0.52 | 0.004 |
| A8. For a child with allergic rhinitis, you will worry that he is more likely to develop asthma than the average child. | 4.14±0.87 | 4.20±0.87 | 0.530 |
| P4. You will supervise and teach your child to avoid contact with allergens as much as possible. | 4.19±0.80 | 4.20±0.83 | 0.961 |
| P5. You will guide your child to take active physical exercise. | 4.43±0.69 | 4.55±0.66 | 0.110 |
| P6. You will guide your child to be physically active. | 4.32±0.69 | 4.47±0.68 | 0.050 |
| P7. You will actively learn about allergic rhinitis. | 3.86±0.97 | 4.00±0.89 | 0.146 |
| P8. You will teach your child about allergic rhinitis. | 3.87±0.98 | 3.88±0.96 | 0.909 |

Supplement Table S2. Pearson correlation analysis of KAP dimension scores

|  | Knowledge | Attitude | Practice |
| --- | --- | --- | --- |
| Knowledge | 1 |  |  |
| Attitude | 0.31 (P<0.001) | 1 |  |
| Practice | 0.477 (P<0.001) | 0.551 (P<0.001) | 1 |

Supplementary Table S3. Scores to the parents’ KAP items according to the children’s age

| KAP items | Score, mean ± SD | | | P |
| --- | --- | --- | --- | --- |
|  | <6 | 7-10 | 11-17 |  |
| K1. Children’s allergic rhinitis, also known as children’s allergic rhinitis, is a non-infectious chronic inflammatory disease of the nasal mucosa after exposure to allergens such as dust mites and pollen. | 1.25±0.67 | 1.34±0.62 | 1.27±0.65 | 0.535 |
| K2. The typical symptoms of allergic rhinitis in children are watery discharge, nasal itching, nasal congestion, and sneezing. | 1.32±0.65 | 1.46±0.65 | 1.36±0.67 | 0.216 |
| K3. When a primary family member has an allergic disease, the child should be managed as a child at high risk of allergic disease. | 0.97±0.76 | 1.2±0.79 | 1.08±0.77 | 0.049* |
| K4. Allergens refer to antigen substances that induce the body to produce allergies, mostly inhaled antigens, with dust mites and pollen being the most common. | 1.27±0.7 | 1.47±0.67 | 1.28±0.7 | 0.050* |
| K5. Inhaled antigens such as mold, animal dander, and cockroaches may also cause allergic rhinitis attacks in children. | 1.24±0.74 | 1.35±0.72 | 1.15±0.75 | 0.091 |
| K6. Do you know about allergen skin testing? | 0.87±0.83 | 1.26±0.75 | 0.92±0.83 | 0.001* |
| K7. Do you know about desensitization therapies (such as dust mite drops) for allergic rhinitis in children? | 0.65±0.77 | 1.04±0.81 | 0.8±0.81 | 0.001* |
| K8. Children with allergic rhinitis and persistent bronchial asthma cannot receive desensitization therapy. | 0.45±0.73 | 0.66±0.85 | 0.67±0.81 | 0.015* |
| K9. Children with allergic rhinitis should avoid contact or minimize contact with allergens. | 1.44±0.72 | 1.6±0.59 | 1.34±0.71 | 0.010* |
| K10. Effective measures to control indoor dust mites: keep the room clean, air circulation, reduce humidity, regularly clean the air conditioning filter; use well-sealed bed boards and pillows; wash bed sheets and pillows with hot water > 54.4 °C; Stay away from stuffed animals, carpets, tapestries, etc. | 1.23±0.74 | 1.39±0.63 | 1.31±0.68 | 0.162 |
| K11. Allergic diseases include food allergies, atopic dermatitis, allergic rhinitis, and allergic asthma. | 1.13±0.75 | 1.33±0.66 | 1.15±0.7 | 0.063 |
| K12. One child may suffer from multiple allergic diseases at the same time. | 1.01±0.81 | 1.15±0.81 | 0.97±0.78 | 0.190 |
| A1. How much do you care about allergic rhinitis in your child: | 4.37±0.64 | 4.6±0.61 | 4.28±0.79 | 0.002* |
| A2. You are concerned that allergic rhinitis may endanger your child’s health. | 4.39±0.71 | 4.71±0.5 | 4.37±0.74 | <0.001* |
| A3. Allergic rhinitis in children will relieve itself, there is no need to pay much attention. | 3.92±1.1 | 3.82±1.18 | 3.87±1.14 | 0.756 |
| A4. Allergic rhinitis in children requires standardized medical visits and treatment. | 4.47±0.67 | 4.62±0.55 | 4.39±0.81 | 0.030* |
| A5. The child’s allergic rhinitis is currently well-controlled and has not adversely affected his daily life. | 3.21±1.14 | 3.13±1.24 | 3.4±1.2 | 0.130 |
| A6. The importance of changing clothes frequently, washing towels and bedding frequently: | 4.69±0.52 | 4.71±0.48 | 4.62±0.57 | 0.263 |
| A7. For exercise, strengthening physical fitness is beneficial to prevent the onset of allergic rhinitis in children | 4.67±0.59 | 4.76±0.45 | 4.69±0.56 | 0.417 |
| A8. For a child with allergic rhinitis, you will worry that he is more likely to develop asthma than the average child. | 4.21±0.83 | 4.4±0.69 | 4.08±0.96 | 0.012* |
| P4. You will supervise and teach your child to avoid contact with allergens as much as possible. | 4.18±0.83 | 4.28±0.77 | 4.17±0.85 | 0.553 |
| P5. You will guide your child to take active physical exercise. | 4.47±0.68 | 4.51±0.65 | 4.58±0.65 | 0.253 |
| P6. You will guide your child to be physically active. | 4.42±0.67 | 4.38±0.74 | 4.47±0.67 | 0.513 |
| P7. You will actively learn about allergic rhinitis. | 3.94±0.92 | 3.99±0.87 | 3.99±0.93 | 0.856 |
| P8. You will teach your child about allergic rhinitis. | 3.72±1.02 | 3.97±0.9 | 3.98±0.92 | 0.018 |

**Supplementary Figure S1.** Confirmatory factor analysis.


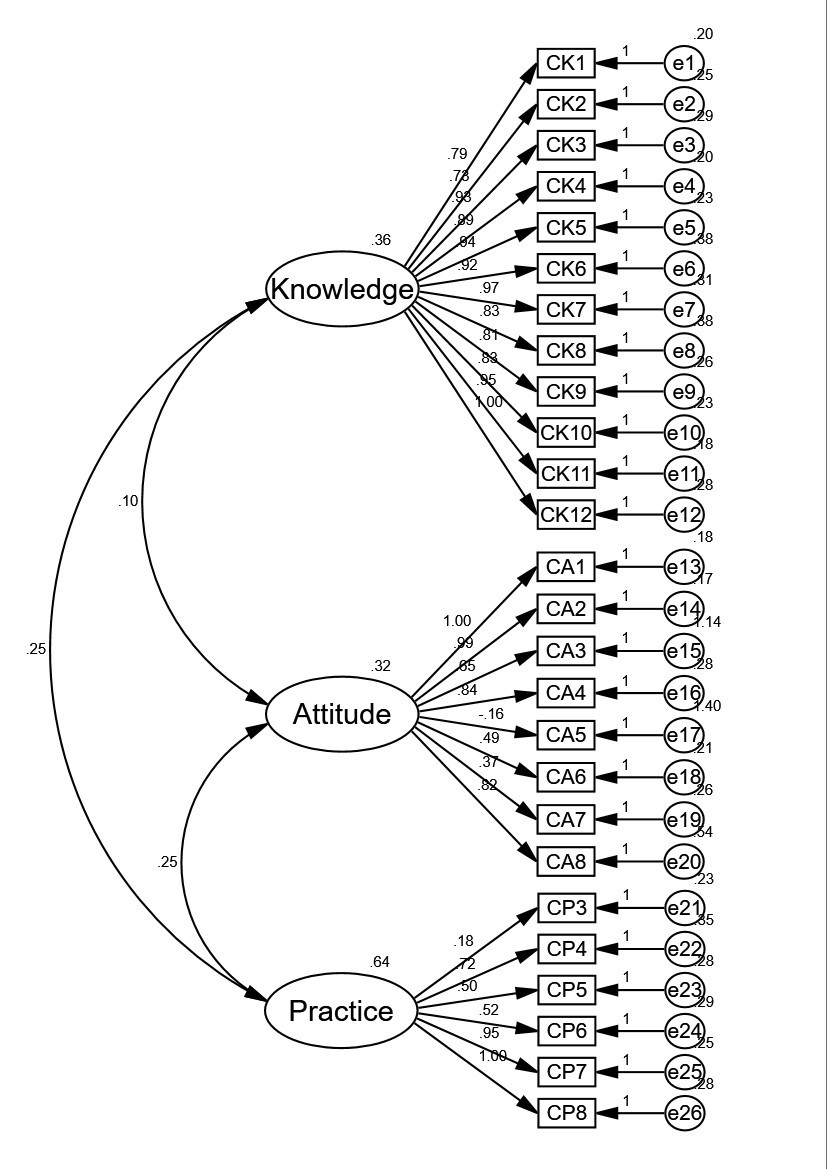

Supplement: Supplementary file 1 — Supplementary Material 1 [file 12889_2024_18581_MOESM1_ESM.docx]
